# Quality assurance for next-generation sequencing diagnostics of rare neurological diseases in the European Reference Network

**DOI:** 10.1038/s41431-024-01639-2

**Published:** 2024-06-05

**Authors:** Aleš Maver, Katja Lohmann, Fran Borovečki, Nicola Wolstenholme, Rachel L. Taylor, Malte Spielmann, Tobias B. Haack, Matthias Gerberding, Borut Peterlin, Holm Graessner

**Affiliations:** 1 Clinical Institute of Genomic Medicine, University Medical Centre Ljubljana, Ljubljana, Slovenia; 2 Institute of Neurogenetics, University of Luebeck, Luebeck, Germany; 3 Department of Neurology, University Hospital Centre Zagreb, Zagreb, Croatia; 4 EMQN CIC, Unit 4 Enterprise Hse, Manchester Science Park, Manchester, UK; 5 Institute of Human Genetics, University of Lübeck, Lübeck, Germany; 6 Institute of Medical Genetics and Applied Genomics, University of Tübingen, Tübingen, Germany; 7 Centre for Rare Diseases, University of Tübingen, Tübingen, Germany

**Keywords:** Neurological disorders, Genetic testing, Genetic testing

## Abstract

In the past decade, next-generation sequencing (NGS) has revolutionised genetic diagnostics for rare neurological disorders (RND). However, the lack of standardised technical, interpretative, and reporting standards poses a challenge for ensuring consistent and high-quality diagnostics globally. To address this, the European Reference Network for Rare Neurological Diseases (ERN-RND) collaborated with the European Molecular Genetics Quality Network (EMQN) to establish an external quality assessment scheme for NGS-based diagnostics in RNDs. The scheme, initiated in 2021 with a pilot involving 29 labs and followed by a second round in 2022 with 42 labs, aimed to evaluate the performance of laboratories in genetic testing for RNDs. Each participating lab analysed genetic data from three hypothetical cases, assessing genotyping, interpretation, and clerical accuracy. Despite a majority of labs using exome or genome sequencing, there was considerable variability in gene content, sequencing quality, adherence to standards, and clinical guidance provision. Results showed that while most labs provided correct molecular diagnoses, there was significant variability in reporting technical quality, adherence to interpretation standards, reporting strategies, and clinical commentary. Notably, some labs returned results with the potential for adverse medical outcomes. This underscores the need for further harmonisation, guideline development, and external quality assessment in the evolving landscape of genomic diagnostics for RNDs. Overall, the experience with the scheme highlighted the generally good quality of participating labs but emphasised the imperative for ongoing improvement in data analysis, interpretation, and reporting to enhance patient safety.

## Introduction

Next-generation sequencing (NGS) approaches are being increasingly adopted as the fundamental approach in the diagnosis of rare neurological diseases (RNDs) [1, 2]. Given the clinically complex and overlapping presentations of neurological diseases and their extensive genetic heterogeneity, NGS-based approaches offer a rapid and cost-effective modality to establish a molecular diagnosis [3]. In particular, comprehensive genomic approaches, including exome and genome sequencing, are increasingly being considered as first-line genetic tests for a variety of RNDs [4, 5].

Although the benefits of NGS in diagnosing RNDs are undisputable, NGS is a complex diagnostic approach consisting of multiple stages that impact the final diagnostic result [6]. Laboratories providing NGS for diagnostic testing use a variety of approaches for the preparation of sequencing libraries, bioinformatic analyses, variant interpretation, and reporting [7]. This diversity also translates into variability in test outcomes, and several studies have demonstrated medically significant interlaboratory variability including differences in test characteristics [8], variant interpretation [9], and clinical reporting [10]. For this reason, there is a need to facilitate the harmonisation of the NGS-based testing process and to establish a mechanism that supports high-quality standards for meaningful NGS-based diagnostic tests for RNDs.

The European Reference Network for Rare Neurological Diseases (ERN-RND) was established in 2017 with the primary aim of improving the care of patients with RNDs in the European Union [11]. As part of this objective, ERN-RND aims to improve the access to high-quality genetic testing in diagnostic laboratories across Europe. This goal depends on ensuring the provision of comparable results of NGS-based tests in RNDs.

To achieve this, the ERN-RND has established an external quality assessment scheme for NGS-based approaches in RNDs´ diagnostics. A pilot quality assessment scheme was performed in 2021 and a second round was carried out in 2022. We present the experiences from these first two external quality assessment schemes and summarise the current state of the provision of NGS-based diagnostics for RNDs in ERN centres across Europe.

## Materials and methods

### Establishment of the scheme

The ERN-RND external quality assessment scheme was established in 2021 in collaboration with the European Molecular Quality Network (EMQN).

The procedures used in the scheme are in accordance with standard procedures employed in the provision of the external quality assessment for molecular diagnostic laboratories [12, 13].

### Scheme operation

In both, the 2021 pilot run and the subsequent 2022 run of the scheme, three DNA samples from patients with RNDs were distributed to participants along with hypothetical case scenarios detailing referral clinical information. The selection of cases in the first year of the scheme was based on a rationale to include straightforward cases to allow appropriate testing and calibration of the marking criteria, without introducing additional complexity in variant interpretation or variant detection. In the second year of the scheme, we have included a case with a deletion to assess the ability of the labs to perform the detection of variation types other than short sequence variation or assess the handling of this limitation of NGS in the diagnostic setting. The case with VCP-linked dementia was introduced to assess the ability of the labs to interpret the results of sequencing in relation to referral clinical symptoms and signs. A negative case was used in both years of the scheme to assess the possible over-interpretation of results by the laboratories. The details of the cases selected for the two schemes are presented in Table 1.

**Table 1:** Presentation of the cases used in the ERN-RND quality assessment scheme.

Season	Case	Diagnosis	Validated result	Outcome
2021	1	Familial Parkinson’s disease	Heterozygous for; NM_198578.4:c.6055G>A p.(Gly2019Ser)	A pathogenic heterozygous variant confirming the presence of *LRRK2*-linked Parkinson disease
2021	2	Spastic paraplegia	Heterozygous for NM_014946.4:c.1291C>T p.(Arg431Ter)	A pathogenic heterozygous *SPAST* variant confirming the diagnosis of *SPAST*-linked spastic paraplegia
2021	3	Amyotrophic lateral sclerosis	No pathogenic variants identified	No molecular cause identified
2022	1	Spastic paraplegia	Hemizygous deletion of NM_000033.4(ABCD1) exons 6-10	A pathogenic deletion in the *ABCD1* gene, confirming a diagnosis of *ABCD1*-linked spastic paraplegia
2022	2	Early-onset frontotemporal dementia with skeletal features	Heterozygous for NM_007126.5(VCP):c.464G>A p.(Arg155His)	A pathogenic variant was identified in the *VCP* gene, confirming a diagnosis of Inclusion body myopathy with early-onset Paget disease and frontotemporal dementia 1
2022	3	Primary brain calcification disorder	No pathogenic variants identified	No molecular cause identified

The laboratories performed the molecular testing, interpretation and reporting and returned the final genetic testing reports.

An accompanying feedback form was provided to the participants to obtain details on the approaches, technology and bioinformatic algorithms used in the diagnostic process.

### Assessment of the scheme results

After completion of the scheme, each returned report was assessed by at least two of three assessors. The assessment scoring was performed in three main domains - genotyping, interpretation and clerical accuracy. Similar to other EMQN-guided schemes [14], a laboratory can achieve a maximum of 2.0 points in each category and deductions are given by the assessors when the laboratory departs from the minimum standards required or makes an error. The deduction depends on the severity of the discrepancy.

We devised NGS-specific assessment criteria to capture the adherence of laboratories to minimal standards in the analytical, interpretative and reporting stages (Table 2).

**Table 2:** Assessment criteria that were developed to ensure the conformance of labs with minimal quality standards of NGS-based test.

**Technical standards**
Provision of unambiguous information on the library preparation and sequencing approach
Provision of the minimum performance criteria of the test (coverage, percentage of targets captured at minimal sequencing depth)
A clear statement of the limitations of an NGS-based test (in particular for negative reports)
**Interpretation standards**
A clear statement of the gene content considered in the interpretation
Declaration of variant assertion criteria used in the interpretation
Declaration of the lines of evidence applied in reaching the variant classification
Appropriate use and weighting of evidence used in variant classification
Adequate evidence used in variant classification
**Reporting standards**
Alignment of the result with the referral medical indication
A clear declaration of the relevance of the finding for the referral diagnosis
Limit the reporting of unrelated or unsupported findings as the principal findings
Provision of condensed and clinically relevant information on the first page of the report

Additionally, criteria that were specific to selected cases were used in the assessment. This included the ability to detect copy number variants (CNV), pertinent to Case 1 in 2022, and the ability of the laboratory to correlate the molecular finding with the referral in its completeness, which was pertinent to Case 2 in 2022.

## Results

### Participation

**Fig. 1:**
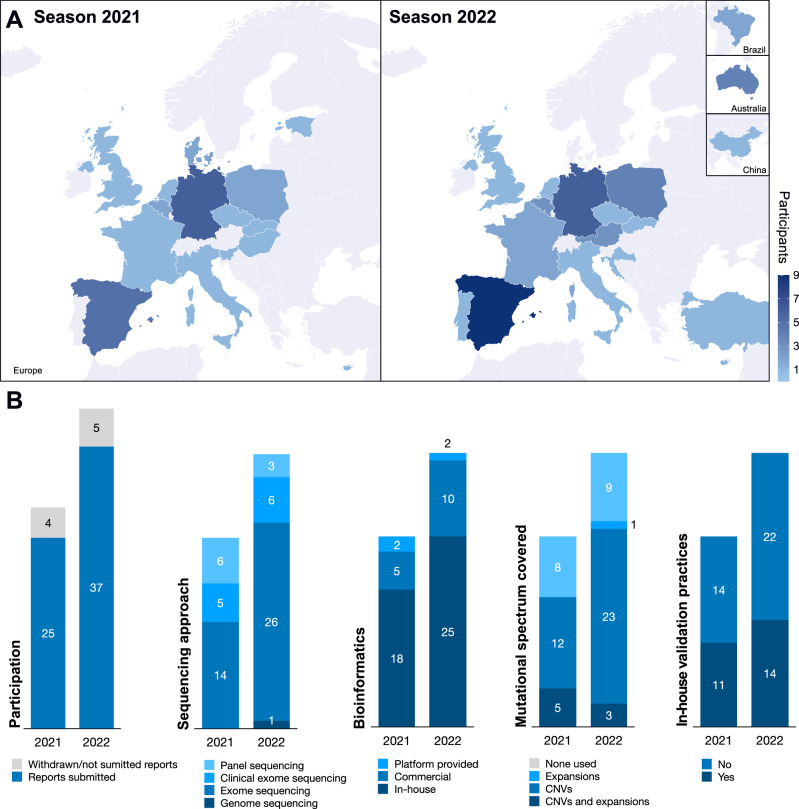
Countries participating in the pilot ERN-RND QA scheme. Figure **A** outlines the number of participating centres from each country, where the darker shade of blue indicates a higher number of participating centres per country. Countries with no participating centres are highlighted in grey colour. Figure **B** represents the number of participating centres in both runs of the scheme. Additionally an overview of self-reported methodological approaches used in diagnostic testing of RNDs is presented for the laboratories that provided this information (including the scope of sequencing, bioinformatics, routine detection of non-SNV genetic variation and use of orthogonal confirmation). All the laboratories participating reported a routine detection of SNVs and indels.

Overall, the scheme received 29 applications in the pilot run and 42 applications in the subsequent year. Of the applicants, 25 and 37 laboratories returned the final reports in the pilot and the subsequent season, respectively. In the pilot run, laboratories from 17 different European countries participated, and laboratories from 18 countries participated in the second year (Fig. 1, panel A).

### Methods and strategies used to diagnose RNDs by participating laboratories

The scheme participants filled out an accompanying feedback form, which provided additional insight into the approaches and methodologies used for diagnostic testing employed by the participating laboratories.

The results indicated the widespread use of comprehensive genomic approaches for diagnostics of RNDs (Fig. 1, panel B). Of the 25 institutions participating in the pilot run, 14 (56.0%) reported using exome sequencing and 5 “clinical” exome sequencing to perform diagnostic testing in the examined cases. The remaining 6 laboratories (24%) reported using gene panel sequencing. The use of comprehensive genomic approaches was even more pronounced in the 2022 season of the scheme, where 33 of 37 (89.2%) laboratories reported using either exome, clinical exome or genome sequencing as the primary diagnostic approach. A majority of labs reported using *in-house* developed pipelines for data analysis (72.0% [18 laboratories] in 2021 and 67.6% [25 laboratories] in 2022).

We have also surveyed the approaches to orthogonal validation of the test results, with 11 laboratories (44.0%) in the 2021 season and 14 laboratories (38.9%) in the 2022 season indicating that they consistently confirm the sequence variants using Sanger sequencing or a related approach. In the 2021 season, five laboratories (20%) indicated a consistent use of complementary methods (ie. MLPA) to capture genomic variation that can be difficult to detect in NGS-based approaches, while this rate was higher in the season 2022 (14 laboratories, 38.9%).

### Laboratory performance

**Fig. 2:**
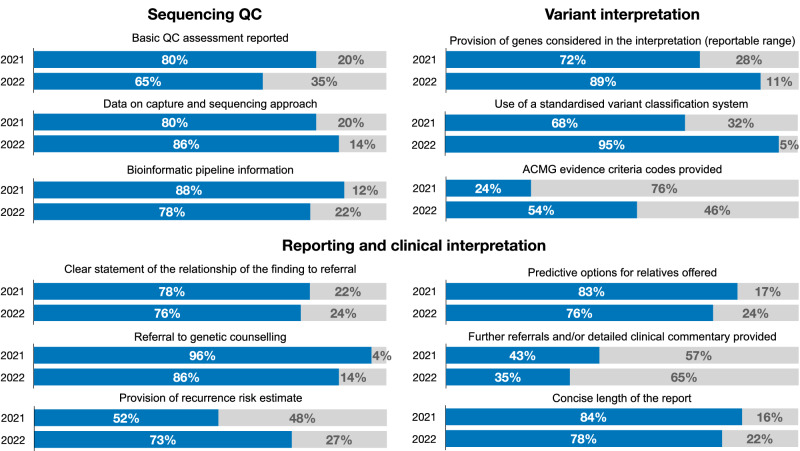
Overview of the outcomes of the 2021 and 2022 ERN-RND scheme results in the domain of provision of NGS-related quality control parameters, quality of variant interpretation and quality of clinical interpretation and reporting. The charts represent the proportion of laboratories that met a criterion surveyed (in blue) versus the rest (in grey).

An overview of the major findings of the scheme is presented in Fig. 2. Of the 25 participants, 23 (88.0%) reported a correct molecular result for all three cases in the pilot run of the 2021 scheme. Two laboratories failed to report the pathogenic *LRRK2* variant in the patient with Parkinson’s disease - in one of the two cases this was because the gene panel investigated did not contain the LRRK2 gene, which is a major gene linked to the referral request of testing for Parkinson´s disease [15]. Additionally, one laboratory failed to detect the pathogenic variant in the *SPAST* gene in the patient with spastic paraplegia, although the gene was targeted.

In 2022, 35 of the 37 participants (94.6%) reported a correct molecular result for all three cases, based on the methods reported to be used. Thus, the success rate slightly increased in the second round of the scheme in 2022 when compared to the 2021 scheme, but there were still two laboratories that failed to provide at least one correct diagnosis. The most challenging case in 2022 was a patient with a hemizygous multi-exon deletion in the *ABCD1* gene. This variant had been included to assess the ability of the participants to routinely detect CNVs in NGS data. Alarmingly, two laboratories failed to detect this CNV despite reporting the use of a CNV calling algorithm. Of note, five laboratories also failed to detect the hemizygous deletion in the *ABCD1* gene but they had clearly indicated that they were not able to detect CNVs with the applied method and indicated, in their reports, the need for additional screening. Thus, these reports were considered correct within the limitations of the test. However, this illustrated that about a fifth (7/37) of the participating laboratories in 2022 were not able to detect this CNV. Of note, hemizygous CNVs in a male are characterised by no calls in the respective region which is a more pronounced change than a heterozygous CNV characterised by a 50% drop in the read depth.

#### Reporting of sequencing quality measures

During the scheme evaluation process, we also monitored the performance of laboratories in terms of reporting of sequencing quality measures, quality of variant interpretation, and reporting (Fig. 2). We assessed the provision of minimal technical specifications of NGS-based tests in participants’ reports. Of the 25 laboratories in the pilot run, five (20.0%) failed to provide the quality parameters required to assess sufficient coverage and the percentage of targeted genomic regions sequenced with acceptable coverage. Furthermore, five (20.0%) labs failed to provide details of the library preparation, enrichment, and/or sequencing protocols used to generate the sequencing dataset. One lab failed to clearly state the genes considered for diagnostic reporting. In the subsequent year, incomplete reporting of quality parameters was more prevalent (13 of 37 labs, 35.1%) and the rate of reporting the essential data on the methodology was comparable to the earlier year, with 6 of 37 (16.2%) labs not reporting the essential information on the methodology used.

#### Variant interpretation

A broad interlaboratory variation with considerable limitations was observed in the performance of variant interpretation. In the pilot run, eight laboratories (32.0%) did not report using an established standard for variant interpretation (i.e. ACMG 2015 guidelines [16]). This portion was similar in the second run (27.0%). While no laboratories made variant classification errors relevant to clinical decision-making in the pilot run of the scheme, two participants incorrectly used evidence in support of the variant’s pathogenicity. One laboratory made a critical interpretation error in the 2022 run of the scheme by reporting a single heterozygous variant in the *FIG4* gene, which is associated with biallelic inheritance, as the cause of clinical presentation described in the referral. Although monoallelic variants in the *FIG4* gene have been associated with amyotrophic lateral sclerosis (OMIM:612577), this specific variant is prevalent in the general population in the heterozygous state. It seems, at best to increase the ALS risk but is not causative on its own. A causative impact of *FIG4* variants in the sense of a monogenic disease have only been demonstrated for biallelic variants in the literature. Further, seven labs of the pilot run (28.0%) did not provide sufficient evidence to support the final classification of reported variants in the 2021 season. This improved in the 2022 season, with only 5 laboratories (13.5%) providing insufficient evidence in favour of variants’ pathogenicity. While only 7 (28.0%) labs provided clear references to ACMG evidence codes used in variant classification in the pilot scheme, we observed an increase in using ACMG criteria in the subsequent run of the scheme (54.0%).

#### Clinical interpretation and reporting

We also noted a wide variability in the style of the participants’ reports and the adoption of best practicers for reporting genetic test results [17, 18]. Eighteen laboratories (72.0%) in the 2021 scheme and 28 laboratories (75.7%) in 2022 provided a clear statement of the clinical relationship of the findings to the clinical presentation described in the referral. The remaining laboratories used variable declarations to describe this relationship, including “possible cause”, “is associated”, “can explain”, “could be compatible”, “may be responsible” and other terms. We also assessed the interpretation of the molecular findings in the clinical context, specifically in the case of frontotemporal dementia and skeletal features due to a specific *VCP* variant (Case 2 in 2022) - here 8 of 37 (21.6%) laboratories failed to make a clear correlation between the observed skeletal features in the patient with the presentation of *VCP*-linked Inclusion body myopathy with early-onset Paget disease and frontotemporal dementia 1.

The reports also differed in terms of the clinical information provided to the referring clinician and/or the patient. In the 2021 season, 12 laboratories (48.0%) provided information on the recurrence risks, 19 labs explicitly stated an option for further testing in relatives (76.0%) and 10 labs (40.0%) provided an extended commentary on the disease course and penetrance. One laboratory (4.0%) did not refer the patient to genetic counselling upon receiving a positive molecular testing report. In the 2022 season, we saw an improvement in the number of laboratories indicating recurrence risk rate (27 laboratories, 73.0%), reporting predictive options for relatives (28 laboratories, 75.7%) and providing detailed clinical commentary of the findings (13 laboratories, 35.1%). Additionally, five laboratories (13.5%) failed to mention referral to genetic counselling in the 2022 scheme run.

In 2021, four labs (16%) returned reports that were considered overly long (over five pages) and too complex by the assessors, whereas this was noted in 8 laboratories (21.6%) in the 2022 season.

One lab reported (likely) benign variants as the principal finding of the diagnostic test. In this regard, one of the variants present in Case 3 in the 2022 scheme has presented a challenge for the laboratories. This patient harboured a *heterozygous* variant in the *FIG4* gene - NM_014845.6:c.122T>C, (p.Ile41Thr), which could be classified as pathogenic. Notably, *biallelic* pathogenic variants in the *FIG4* gene cause neuropathy, a condition distinct from a brain calcification disorder, which was the diagnostic referral in this case. Nevertheless, one laboratory reported this finding as the cause of the disorder in this patient despite the mismatch between the clinical presentations.

## Discussion

We report on the establishment of an external quality assessment scheme for NGS-based testing of RNDs in ERN-RND and present the outcomes of the first two runs of the scheme operation in 2021 and 2022. Results of the assessment indicated a relatively high accuracy and reproducibility of genotyping results across participating laboratories but also false-positive and false-negative reports. In addition to the diagnostic outcome, the content and presentation of the results in the report was evaluated. For this, we observed broad variation in the completeness of reported quality metrics, methodological information provided, quality of variant interpretation and reporting by participating laboratories. The summary of the principal observations during the provision of the scheme is presented in Table 3.

**Table 3:** Overview of the primary challenges and recommendations to harmonise reporting of the NGS-based diagnostic tests for rare neurological disorders.

Quality measure	Challenges observed during the scheme provision	Recommendations for harmonisation
Information on the details of the test performed	A proportion of the laboratories failed to clearly present the sequencing platform, or the capture reagent used to enrich the sequencing targets. Information on the bioinformatic software used to process data is not reported by some laboratories.	The sequencing platform and the capture kit along with the version should be provided in the report. If an in-house capture is used, the laboratory should enable the access to the list of targeted regions in the genome. The essential elements of bioinformatic pipeline should also be included in the report. Especially, the information pertaining to detection of non-SNV variation should be provided, for example – details on detection of copy number variation or repeat expansions.
Provision of quality parameters of the sequencing experiment	The NGS experimental quality parameters are not reported consistently across laboratories.	Report the estimate of depth of coverage and the percentage of target regions covered at a defined depth threshold for variant calling
Definition of the reportable range	The genes and the variation spectrum assessed is inconsistently reported. There are significant discrepancies in the sets of genes that laboratories consider in the interpretation process. An example is the failure to include recently reported genes in the reportable range.	The laboratories should clearly report the genes assessed in their analysis and indicate the spectrum of genetic variation assessed in the test. The sets of genes should be regularly updated. Alternatively, regularly curated and versioned virtual panels may be used (for example, the PanelApp resource).
Adherence to variant interpretation standards	Variable use and reporting of pre-defined variant interpretation standards is observed.	The laboratories should provide the information on the variant interpretation standard use. If an in-house variant classification system was used, it should be accessible upon request.
Reporting evidence to support variant pathogenicity assertion	Several laboratories do not clearly present the lines of evidence that support the final variant classification	The lines of evidence should be clearly and distinctly presented in the variant interpretation. If the ACMG guidelines are used, reporting of evidence codes and the assigned evidence strengths is strongly advised. The laboratories should include information on the specifications of the variant evidence or gene-specific classification system modifications, if they are used.
Clinical interpretation	The relevance and association of the reported finding to the clinical referral is not presented consistently	The laboratories should clearly indicate the relevance of primary findings in terms of their association with referral indication and report if a finding is consistent with the referral clinical presentation.
Clinical guidance	There is a variability in provision of clinical commentary and guidance in the reports	The necessity of genetic counselling should be consistently indicated on the reports. Indication of the mode of inheritance should be indicated for primary findings, and if possible, an assessment of recurrence risk. In case of significant limitations of NGS approaches in detecting a particular disorder (for example, when reporting a negative result for a referral primarily associated with repeat expansions), this limitation should be highlighted in among the primary conclusions of the test. If further genetic testing is needed to clarify the diagnostic relevance of the finding (for example, a segregation analysis), this should also be advised in the clinical report.
Report format	Several laboratories provide lengthy reports that require significant effort to extract the relevant information.	Despite the complexity of NGS-based genetic testing, it is recommended to state the principal outcomes and conclusions of the testing concisely on the first page of the report

While NGS is generally considered an improvement compared to classical Sanger sequencing approaches in terms of throughput and diagnostic yield, especially in highly heterogeneous disorders such as rare neurological disorders, it also has several properties that need to be taken into consideration when it is applied for diagnostic purposes. In both runs of the ERN-RND quality assessment scheme, we observed that a number of labs did not report basic information on the laboratory protocol and/or quality metrics of the NGS-based test that would allow an accurate evaluation of the limitations of the test performed and identify gaps with insufficient coverage. Principal test performance characteristics (including sequencing depth and percentage of gene target sequenced at a coverage minimum) were also not consistently reported by the participating laboratories. There is a wide variability of exome sequencing capture methodology employed and this significantly impacts the sensitivity of the test in specific genomic regions [19]. The frequent omission of these details has already previously been documented for diagnostic NGS reports [10].

As NGS-based testing typically investigates large sets of genes, clinical interpretation of the resulting comprehensive datasets is challenged by the large number of variants detected, which therefore increases the possibility of misclassification. For this reason, strict adherence to up-to-date variant interpretation standards is essential in molecular genetic testing, especially in rare disorders for which only a limited amount of data is available. Mixed adoption of currently valid standards in interpretation was observed among labs participating in the ERN-RND EQA scheme. Furthermore, we observed worrying inconsistencies in the application of lines of evidence for variant classification and a number of labs did not provide sufficient evidence to support the final variant classification (ie. basing the variant classification on the ClinVar entry, including low-confidence variants (one-star variants) without providing additional lines of evidence supporting pathogenicity). Correct use and application of evidence for variant pathogenicity assertion are essential for less frequently reported and less characterised variants where wide variability in interpretation is anticipated. Despite the widespread use of the ACMG criteria for variant interpretation [16] by the laboratories that did report using a standardised system for variant interpretation, only a minority of laboratories provided specific evidence codes that would make the applied criteria and their strength verifiable and allow for easy updating.

Apart from the general recommendations for diagnostic use of NGS [17], there are currently no specific guidelines on the minimal required content and recommended format of NGS-based reports. Accordingly, we also observed a large variability in the format in contents of the sequencing reports returned by participants in the quality assessment schemes. Particular variation was observed in the declaration of the clinical relevance of the finding to the referral clinical presentation, where various and often unclear wording was used to describe the relevance of the findings. Another notable difference among the reports was observed in the amount of clinical guidance, clarification and advice offered to the report recipient - either the referring medical specialist or the patient. The length of the report was also variable and some of the reports were excessively long, making them difficult to follow and understand the relevant clinical information.

A comparison of the outcomes in the pilot versus the second run of the scheme demonstrated results, with similarly high molecular detection rates but large differences in the variant interpretation, data analysis and reporting of the results. We observed a trend towards an increasing adoption of accepted variant assertion guidelines, increasing transparency in provision of evidence to support variant classifications and provision of relevant gene content analysed in the test.

Furthermore, the scheme provides a valuable insight into the methods and approaches used for NGS-based diagnostics of RNDs in ERN centres. We observed that the majority of the ERN-RND laboratories reported using comprehensive genomic testing approaches. This was despite the fact that clinical referrals warranted investigation of relatively small sets of genes, i.e. the number of genes currently linked to typical Parkinson’s disease comprises fewer than 20 genes [20]. This can likely be attributed to the fact that laboratories are resorting to using expanded genomic tests coupled with virtual panel analysis rather than utilising targeted sequencing panels for hundreds of different diseases with the constant need to update such panels.

Additionally, the scheme sheds light onto shifting diagnostic practices - we observed that less than half of laboratories perform confirmatory testing of sequence variants reported as a diagnostic finding, which is likely due to recent studies demonstrating that this is not necessary for high-quality variants. We have noted that only a few laboratories reported that they consistently perform complementary analyses, such as Sanger sequencing to cover gaps or MLPA-based detection of exonic deletions/duplications, in addition to sequencing. The effect of this shift in the genetic testing landscape may have an impact on the outcome of genetic testing, therefore its impact should be observed and measures should be taken to ensure comparable detection sensitivity and specificity in genetic testing of RNDs. The approaches to quality assessment should be continuously tailored to account for these changes in approaches to genetic testing.

A notable feature of RNDs genetic testing stems from the notion that these disorders are genetically extremely heterogeneous and are also associated with a variety of mutational mechanisms, that may not be readily detectable by standard bioinformatic work-up of targeted short-read NGS-based approaches, including repeat expansions, CNVs and sometimes recurrent non-coding pathogenic variants. Therefore, an appropriate interpretation and reporting of results considering these limitations should be ensured by the ERN-RND quality assessment scheme. Recent publications report an increasingly expanding mutational spectrum detectable by NGS-based genetic testing [21, 22]. Although initially, NGS has been primarily aimed at detecting sequence variants, attempts have been undertaken to detect CNVs [22, 23], repeat expansions [24], mitochondrial variants (if included in the enrichment) [22] and uniparental disomy [25]. While targeting this broad spectrum of variant types has consistently been shown to improve the diagnostic yield of genetic testing, these approaches are performed using a broad spectrum of bioinformatic pipelines with a wide variation in their sensitivity, specificity and consistency of use in molecular diagnostic labs. With the inclusion of a case with a hemizygous deletion in the *ABCD1* gene, we were able to estimate the rate of adoption of CNV calling algorithms in the diagnostic pipelines of laboratories. Here almost a fifth (18.9%) of the labs failed to detect the hemizygous deletion in the *ABCD1* gene, which is characterised by no reads over four exons of the gene. As we recognise that laboratories variably use detection of CNVs, failure to detect the ABCD1 deletion was not regarded as a critical error for laboratories that did not report using CNV detection algorithms. It is nevertheless expected that the hemizygous loss of a substantial part of the gene (gap in coverage) should prompt a targeted revision of the coverage of this gene and therefore lead to the identification of the possible deletion. Alarmingly, two of the laboratories that failed to detect this deletion indicated the use of a CNV calling algorithm in the report. This suggests a notable source of variability in the use and accuracy of extended algorithms for the detection of genetic variation in addition to single nucleotide variants (SNVs) and in insertion-deletion variants (indels). To date, no repeat expansion or non-coding variant has been included in the schemes and thus, no statement can be given on the number of laboratories able to detect these types of variants. However, it can be speculated that it will be lower than the number for successful CNV detection. Therefore, the application of these approaches should also be monitored as a part of the external quality assessment in the ERN-RND scheme.

The wide variability in the results of the ERN-RND scheme across participating labs highlights the urgent need to initiate activities to harmonise the NGS-based testing across (European) laboratories and stresses the need for clear and direct expert opinion-based recommendations and/or best practice guidelines for reporting results of NGS-based testing for RNDs. Through continuous monitoring in the external quality assessment developed by ERN-RND and the development of expert opinion-based recommendations and/or best practice guidelines tailored for the specific genetic context of RNDs, we should promote consistent adherence to diagnostic test standards, variant interpretation and reporting standards. The results of this assessment represent a valuable basis for selection of topics in development of best practice guidelines and recommendations. We present broad recommendations to address the challenges and interlaboratory variability in NGS-based testing for RNDs in Table 3, based on the discrepancies noted during the scheme. These represent a starting point to improve harmonisation of the NGS-based testing for rare neurological disorders and extend the previously published guidelines for reporting results of diagnostic genomic testing [18]. As genetic testing progresses towards the widespread use of genome sequencing in the diagnostic evaluation of RNDs, ensuring high quality of interpretation and reporting is essential to improve access to genetic testing of sufficient quality across different laboratories.

As of January 1st 2022, ERN-RND is composed of 68 national centres of expertise from 25 European Union member states. Expanding the participation in the NGS EQA to these clinical centres will significantly increase the number of participating laboratories. In combination with the development of expert opinion-based recommendations and/or best practice guidelines for NGS-based testing in RND, this might lead to a quality-assured harmonisation of how NGS-based testing is being applied for RND patients in Europe and, thus, to an improvement of diagnostic care provision.

## Data Availability

The datasets generated during and/or analysed during the current study are available from the corresponding author on reasonable request.
